# Stability, orientation and position preference of the stem region (residues 689-703) in Hepatitis C Virus (HCV) envelope glycoprotein E2: a molecular dynamics study

**DOI:** 10.12688/f1000research.2-64.v2

**Published:** 2013-07-02

**Authors:** Rahmad Akbar, Siti Azma Jusoh

**Affiliations:** 1 Bioinformatics Unit, Universiti Teknologi MARA (UiTM), Bandar Puncak Alam, 42300, Malaysia

## Abstract

Envelope glycoproteins of Hepatitis C Virus (HCV) play an important role in the virus assembly and initial entry into host cells. Conserved charged residues of the E2 transmembrane (TM) domain were shown to be responsible for the heterodimerization with envelope glycoprotein E1. Despite intensive research on both envelope glycoproteins, the structural information is still not fully understood. Recent findings have revealed that the stem (ST) region of E2 also functions in the initial stage of the viral life cycle. We have previously shown the effect of the conserved charged residues on the TM helix monomer of E2. Here, we extended the model of the TM domain by adding the adjacent ST segment. Explicit molecular dynamics simulations were performed for the E2 amphiphilic segment of the ST region connected to the putative TM domain (residues 683-746). Structural conformation and behavior are studied and compared with the nuclear magnetic resonance (NMR)-derived segment of E2 ( 2KQZ.pdb). We observed that the central helix of the ST region (residues 689 - 703) remained stable as a helix in-plane to the lipid bilayer. Furthermore, the TM domain appeared to provide minimal contribution to the structural stability of the amphipathic region. This study also provides insight into the orientation and positional preferences of the ST segment with respect to the membrane lipid-water interface.

## Introduction

Envelope glycoproteins E1 and E2 are essential for the initial binding and internalization of Hepatitis C Virus (HCV) into host cells. Both glycoproteins have been shown to interact as a non-covalent heterodimer during biosynthesis ^1^. Several conserved charged residues located in the transmembrane (TM) domains of E1 and E2 were shown to function not only as membrane anchors, but were also essential for dimerization, endoplasmic reticulum retention and viral envelope formation ^2, 3^.

In our previous studies, we demonstrated that the unfolding behavior of the E2 TM helix monomer was attributed to the charged Asp728, which was located in the hydrophobic core. The main contribution of Asp was postulated to be located at the helix-helix interface and involved the formation of a salt bridge with the Lys of the E1 envelope glycoprotein ^2^. The ion-pair interaction of the E1–E2 heterodimer was captured in the molecular dynamic (MD) simulation studies based on the model that placed the charged Asp and Lys at the helix-helix interface ^4, 5^.

E2 envelope glycoprotein is known to be required for interactions with cellular receptors involved in endocytosis and membrane fusion ^6– 8^. E2 is composed of domain I-III, followed by the ST region and the TM domain ^7^. Recent studies based on circular dichroism (CD) and nuclear magnetic resonance (NMR) spectroscopy revealed that the soluble region located adjacent to the TM domain of E2 was involved in the initial virus entry. This highly conserved ST region was shown to fold as a helix upon membrane binding ^9^.

In this work, we carried out MD simulations for three E2 structures: (1) a model generated by the I-Tasser server, (2) an ideal helix model and (3) an NMR derived structure of the E2 segment ( 2KZQ.pdb). The first two models include the TM domain of E2 but the TM domain is not present in the NMR structure. We observed consistent structural stability in the ST region amphipathic segment (residue 689–703) across all simulations suggesting that the contribution of the TM domain to the segment structure stability is minimal. In addition, we demonstrated the orientation and positional preferences of this amphipathic segment.

## Methods

### Input structure preparation

The protein sequence of HCV E2 genotype 1a (H77 strain) (Uniprot ID P27958) used to prepare the models for MD simulations was obtained from UniProtKB/Swiss-Prot database ( www.uniprot.org) ^10^. The following is the sequence segment of HCV E2 used to prepare the first model for this work, referred to as an ideal helix model: ^683^PALSTGL ^690^IHLHQNIVDV ^700^QYLYGVGSSI ^710^ASWAIKWEYV ^720^VLLFLLLADA ^730^RVCSCLWMML ^740^LISQAEA. The ideal helix model was generated using Pymol ( http://pymol.sourceforge.net) by orienting the ST segment (residue 683–714) perpendicular to the TM domain (residues 715–746). The protein structure images in this work were prepared by the Pymol program. The I-Tasser webserver ^11^ was used to generate the second model. The complete sequence of E2 was submitted to the I-Tasser server. Five models were generated and the model with a low root mean square deviation (RMSD) with the available NMR structure ( 2KZQ.pdb) and consisting of a helical TM domain was selected. Only the same segment as examined with the ideal-helix model was used for further MD simulations. The first model of the E2 NMR ensemble (PDBID: 2KZQ) retrieved from the Protein Databank (PDB) ^12^ was used in the third simulation system. This E2 protein segment was based on the HCV genotype 2a (JFH-1) (Uniprot ID Q99IB8) ^9^.

### System preparation

Pre-equilibrated dipalmitoylphosphatidylcholine (DPPC) lipid bilayer was retrieved from the web of Prof. Tieleman ( http://moose.bio.ucalgary.ca/). Peptide orientation in DPPC lipid bilayer was done by aligning the hydrophobic belt of the peptide, parallel to the membrane plane using LAMBADA ^13^. The optimal number of overlapping lipid molecules was subsequently calculated and removed followed by lipid expansion (inflation) and alternating twenty steps of deflation and energy minimization to allow the peptide to be embedded within the bilayer using inflateGRO2 ^13^. A short 100 ps energy minimization was employed to relax possible steric conflicts. Ions and counter ions were added to neutralize the system followed by 20 ns position-restrained simulation allowing the bilayer to re-equilibrate around the protein. Production MD simulations were carried out for 100 ns in the I-Tasser model, and 20 ns for both the ideal helix model and NMR structure (2KZQ.pdb).

### MD simulations

The DPPC lipid bilayer interactions were described using the Berger force-field parameters ^14^. The TM helices were modeled with the united atom force-field GROMOS96 53a6 ^15^. Simulations were performed with the Gromacs 4.5.5 package ^16^ using 2-fs time steps. Periodic boundary conditions were used in all directions. Bonds to hydrogen atoms were constrained using the LINCS algorithms ^17^. For the short-range *van der Waals* interactions, a cut-off distance of 1.0 nm was used. The long-range electrostatic interactions were treated using the particle mesh Ewald (PME) method with a grid spacing of 0.12 nm and cubic interpolation. The non-bonded pair list was generated every 10 steps with a cut-off of 1.0 nm. Water, lipid and peptide systems were coupled separately to temperature baths with 323 K for the DPPC using the Berendsen algorithm with a time constant of τ _T_ = 0.1 ps ^18^. To maintain constant pressure, semi-isotropic coupling was employed separately for the lateral and for the normal directions with Berendsen weak coupling and a τ _p_ = 1 ps time constant. The compressibility was set to 4.5 × 10 ^-5^ bar ^-1^ ^18^.

Analyses of the trajectories were primarily performed with tools included in the Gromacs 4.5.5 suite ^16^. RMSD analyses were based on the coordinates of all atoms of the peptides. The bilayer thickness was measured by averaging the distances between lipid headgroups in the upper and lower leaflets of the lipid membrane with the tool GridMAT-MD ^9^.

## Results and discussion

### Stability of the amphipathic segment

**Figure 1.**
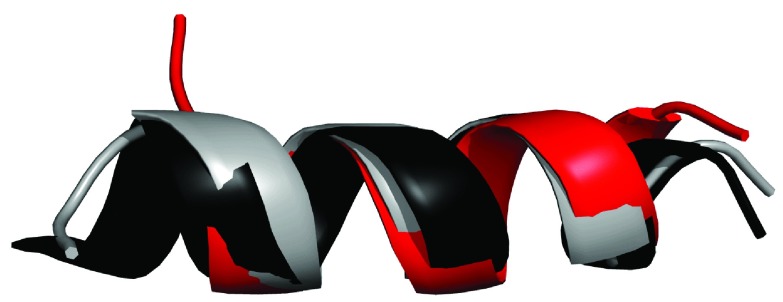
Superimposition of the amphipathic region of E2 (segment 689 to 703) in three molecular dynamics simulations. The most stable helical region of the E2 amphipathic segment during molecular dynamics simulations. 2KZQ in red, I-Tasser model in black and ideal helix model in gray.

**Figure 2.**
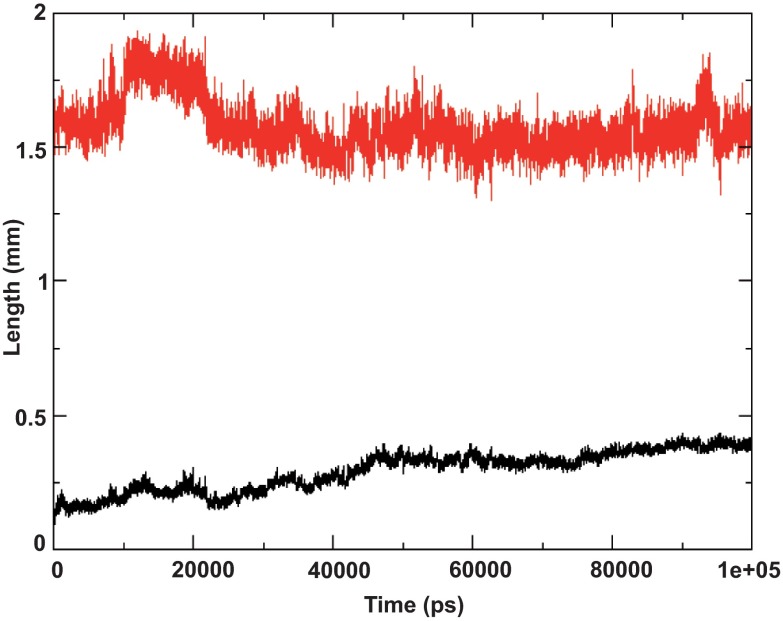
Helix length and RMSD of I-Tasser model. Both amphipathic regions and transmembrane domain located in the hydrophobic core of the bilayer. Length of helix (residues 689–703) is plotted in red, root mean square deviation of the same residues with respect to the starting structure is plotted in black.

**Figure 3.**
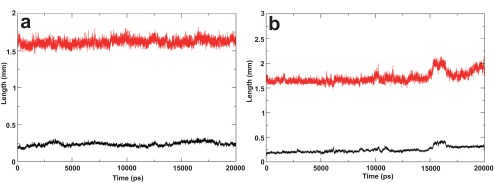
Helix length and RMSD of the ideal helix model and 2KZQ structures. ( **a**) Molecular dynamics simulation of the ideal helix model. The amphipathic segment was positioned in water-lipid interface and the transmembrane domain was oriented in the hydrophobic core of the bilayer. The length of the helix (segment residue 689–703) is plotted in red, root mean square deviation (RMSD) of the same residues with respect to the starting structure is plotted in black. ( **b**) Molecular dynamic simulation of the nuclear magnetic resonance derived structure (2KZQ.pdb). 2KZQ was positioned in the water-lipid interface. The length of the helix segment is plotted in red; RMSD of the same residue with respect to starting structure is plotted in black.

A stable helical conformation of the E2 ST region (residue 689–703) was consistently observed with some uncharacteristic spikes in the early stages and towards the end of the MD simulation of the I-Tasser model and the NMR derived structure (2KZQ.pdb), respectively. RMSD of this amphipathic segment was also consistently observed to be progressing within the commonly accepted 2 Å range for the ideal helix and NMR structures throughout the simulations. On the other hand, the I-Tasser model showed subtly higher RMSD progression over the simulation time ( Figure 1, Figure 2 and Figure 3a, 3b).

Secondary structure stability observed in these three contrasting simulation systems can be attributed to the amphipathic nature of the residues allowing the helix to retain its structure on both the hydrophobic core of the lipid bilayer and the hydrophilic environment of the solvent. Furthermore, secondary structure analyses done by Albecka *et al.* predicted that the amino acid segment 689–703 consists of more than 50% helix. In addition to that, consistent results were shown from CD spectra and NMR with 50% 2,2,2-trifluoroethanol (TFE) environment, which both detected the presence of helical segments in the E2-ST region. The most amphipathic helix characteristics are displayed in the E2-ST segment 687–703. Based on the profile, Albecka *et al.* speculated that this segment is a helix upon membrane binding ^9^. Interestingly, the presence of the TM domain in our simulation does not appear to contribute significantly to the helix stability of the amphipathic region.

The helical length of this region does not vary much between the I-Tasser and the ideal helix models ( Figure 2 and Figure 3a), which include both the ST region and TM domain, compared with the 2KZQ.pdb ( Figure 3b) which does not include the TM domain. This observation suggests that lipid-peptide interactions play a larger role in stabilizing the secondary structure of this amphipathic segment compared with the TM domain. These data provide evidence of the contribution of lipid to structural stability modulation and are in good agreement with the hypothesized lipid and/or protein contribution to structural stability ^9^. The higher RMSD value observed in the I-Tasser model simulation was well anticipated and was mainly attributed to the relative positioning of the ST region, which was sandwiched in between lipid leaflets, forcing the segment to reorganize its structure conformation and having only a minimal effect on the helical integrity of the secondary structure. Examining this reorganization further by monitoring the distance of the amphipathic segment to lipid leaflets led to another interesting observation described in the next section of this article.

### Orientation and positional preference of the amphipathic segment

**Figure 4.**
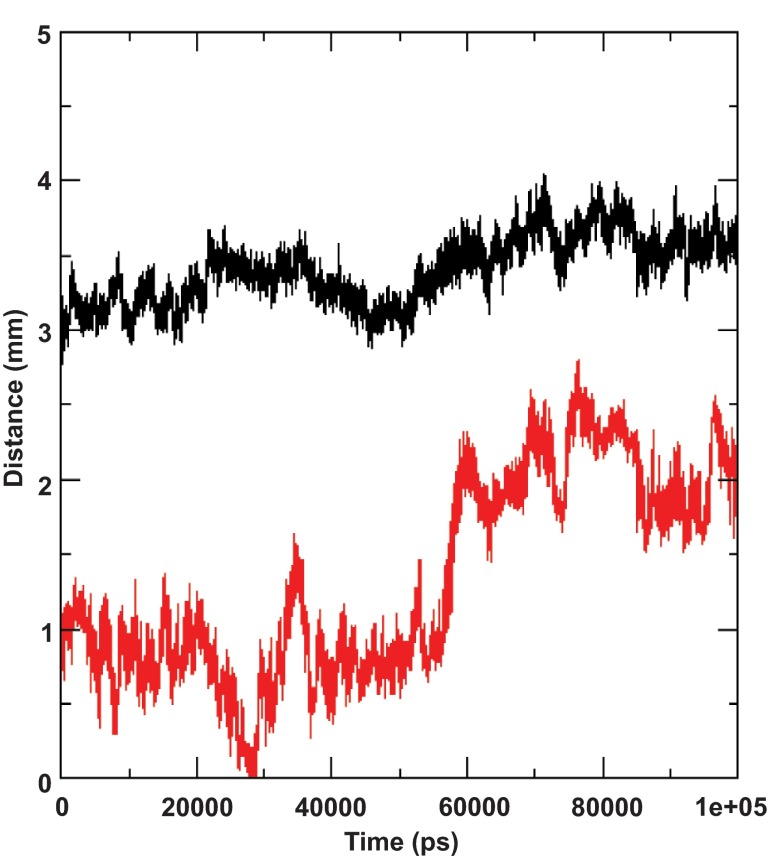
Distance between the helix (residues 689–703) and lipid leaflets during molecular dynamic simulation of the I-Tasser model. The distance to the upper lipid leaflet is plotted in red and lower leaflet is plotted in black.

**Figure 5.**
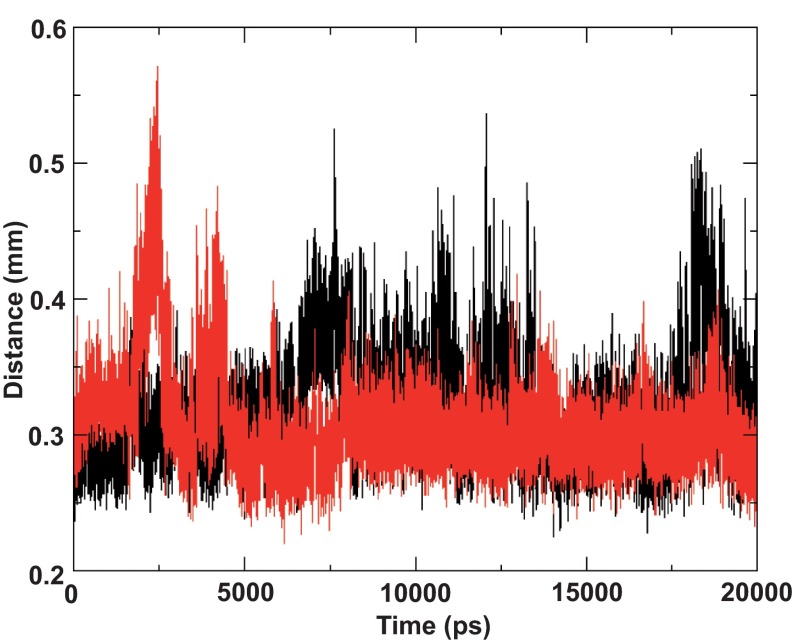
Distance of the amphipathic segment to the lipid leaflets. The 2KZQ is colored in black while the ideal helix model is colored in red. Amphipathic residues remain within 4 Å from the phosphate head group throughout the simulations.

**Supplementary Figure 1.**
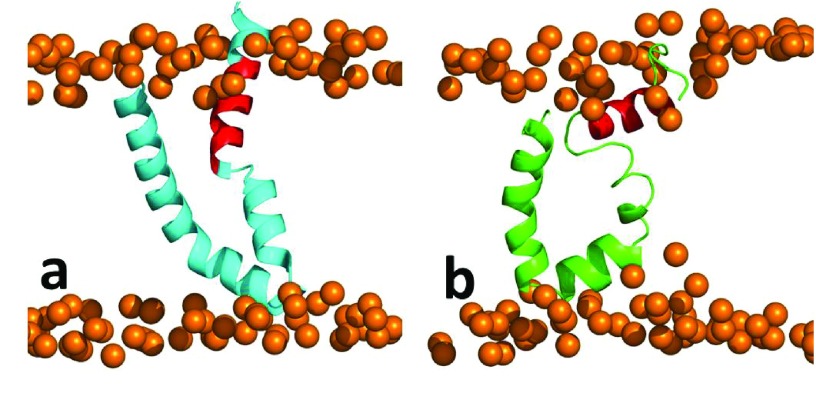
Molecular dynamic simulation of I-Tasser model. ( **a**) Initial starting (cyan) and ( **b**) 100 ns snapshots (green) of molecular dynamic simulations. Segment residues 689–703 are colored in red.

Monitoring the movement of the amphipathic segment during the simulations led to another interesting observation. Segment of residues 689–703 in the I-Tasser model appeared to move towards the hydrophobic core of the lipid bilayer as depicted by steady progression in the distance to both the upper and lower lipid leaflets depicted in Figure 4. This reorganization is surprising and interesting because we would have previously assumed that the amphipathic region exposed to the solvent would hold the structure steady, despite some part of the amphipathic region being initially vertically positioned in the hydrophobic core of the lipid ( Supplementary Figure 1). In addition, given the amphipathic nature of the residues in this segment, one could postulate that the residues would remain in this position. However, over the period of the simulation the segment reoriented by moving away from the lipid leaflets, while at the same time retaining structural integrity. The structural stability of the segment, as discussed in the previous section of this article, is attributed to the amphipathic nature of the residues but this does not explain the movement towards the hydrophobic core of the lipid. The systematically orchestrated movement towards the hydrophobic core of the lipid leaflets indicates a strong orientation preference of the amphipathic segment, which in this specific case was parallel to the lipid leaflets. We then monitored the segment movement relative to the lipid leaflets with the other two simulations (the ideal helix model and the 2KZQ structure). The segment was initially positioned horizontally to the lipid leaflets. The results showed that the distance of the amphipathic segment in both simulations was consistently within 4 Å to the lipid phosphate head group throughout the simulation ( Figure 5). These data further clarify the orientation preference of the amphipathic segment with respect to the lipid leaflets and suggest that the residues are positioned at the lipid-water interface in a very stable manner. Interestingly, Albecka *et al.* ^9^ speculated that these residues could have an in-plane topology or orientation and suggested that the ST region would ideally be positioned at the membrane lipid-water interface, which is again in agreement with our data ^9^.

### Residue contributions and relevance

**Supplementary Figure 2.**
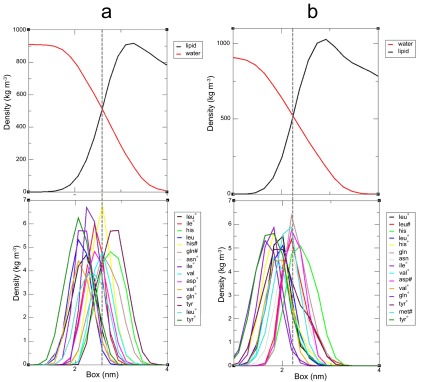
Residue distribution at the lipid-water interface. ( **a**) Ideal helix model. ( **b**) 2KZQ NMR structure. Residues denoted with asterisk (*) and hash (#) are distributed in water (hydrophilic environment) and lipid-water interface, respectively. Residues without denotation are distributed in the lipid bilayer (hydrophobic environment).

**Supplementary Figure 3.**
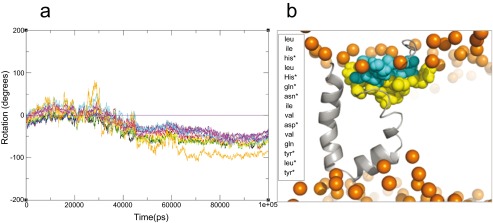
Residue rotation of I-Tasser model. ( **a**) Synchronized rotation was observed, convergence was also observed at around 50 ns and the remaining of the simulation time. ( **b**) Residues pointing to hydrophilic regions are denoted with asterisks and colored in cyan and residues pointing to hydrophobic regions are colored in yellow.

Having observed a clear preference of amphipathic segment orientation in the lipid bilayer environment, it was also pointed out to us that it would be desirable and interesting to look into residue contribution on this behavior. We then plotted the residue distribution in the lipid water interface for the 2KZQ structure and ideal helix model ( Supplementary Figure 2) to identify specific residues positional preference in the lipid water interface. As depicted in Supplementary Figure 2, a clear residue preference in lipid-water interface was not observed. We found polar and charged residues distributed in both hydrophilic and hydrophobic regions of the interface; a similar observation was also found for non-polar residues. This result indicates the overall contribution of the residues played more roles in the stability and positional preference of this segment in lipid-water interface superseding any individual residue contribution. Characterizing the residue distribution in our I-Tasser model would not provide information on the residues preferences since the segment is situated exclusively in a hydrophobic environment. To get some insight on this segment, residue rotation analysis of this segment coupled with qualitative visualization of polar, nonpolar and charged residues was carried out ( Supplementary Figure 3). Synchronized rotation of the amphipathic segment was observed and convergence was captured after 50 ns and for the remaining period of the simulation. In contrast to the ideal helix model and 2KZQ structure simulations, a clear preference to hydrophilic and hydrophobic regions was observed. Polar and charged residues were oriented towards the hydrophilic region and non-polar residues were oriented towards the hydrophobic region. We also noticed that convergence in rotation on 50 ns ( Supplementary Figure 3) coincide nicely with a major shift in distance profile ( Figure 4), which also started at around 50 ns and stabilized at around 70 ns. This indicates that the reorganization of this segment correlates tightly to the residues’ preference towards hydrophilic or hydrophobic environments. It is interesting to highlight that these residues’ preference to hydrophilic and hydrophobic regions in lipid-water interface (ideal helix model and 2KZQ structure) appears to be loosely correlated (no clear preference observed) in contrast to the residues’ preference in environments exclusively on lipid (I-Tasser model) where a clear preference was observed. Due to the fact that the movement of the I-Tasser model residues is synchronized, we found no evidence to substantiate major contribution of individual residues to this structural rearrangement. Overall hydrophilic and hydrophobic residue contribution to the segment reorientation and reorganization is further highlighted.

### Limitations and future work

An orientational preference parallel to the lipid leaflets is a common feature of amphipathic helix segments. Parallel orientation of amphipathic segments has been characterized in experimental works ^20^. Amphipathic segments have also been observed to only minimally penetrate and contribute to lipid bilayer perturbation ^21, 22^. In addition to that, lipid shape has been demonstrated to contribute to amphipathic segment topology in the lipid interface ^23^. Taking all this information collectively, the amphipathic segment of residues 689–703 is anticipated to be positioned stably in the lipid-water interface in a parallel topology with respect to the lipid leaflets. In line with this anticipation, the 20 ns simulations of two of our structures (ideal helix model and 2KZQ) demonstrate a consistent helical form oriented parallel to the lipid leaflet throughout the simulation period. Interestingly, our third simulated structure (I-Tasser model) demonstrated a clear preference to parallel orientation despite the fact that its initial amphipathic segment configuration was perpendicular to the lipid leaflet. We decided to subject the I-Tasser model to longer simulation (100 ns) compared to the other two structures (20 ns) mainly due to the position of its amphipathic segment. The I-Tasser model positioned the amphipathic segment sandwiched in the bilayer leaflets, in contrast to the commonly assumed position in the membrane interface. On the other hand in the ideal helix and 2KZQ models it is readily positioned at the membrane interface. True to our expectation, meaningful observations such as amphipathic segment reorganization only started to be captured after 70 ns. This highlights the importance of optimum conformational space exploration in the simulation. Despite the consistent stability observed throughout our ideal helix model and 2KZQ structures, a longer simulation time is desirable to further reaffirm the observation. Simulation of the amphipathic segment in the water-only environment is also desirable to elucidate the segment behavior. These will be added as part of the future agenda of this work.

## Conclusion

In this study, the atomistic MD simulations provide insightful structural data for the E2 segment. The amphipathic segment of E2 was able to remain as a stable helix in a lipid bilayer environment even without the respective TM domain. The results also describe orientation and positional preferences of the amphipathic segment in relation to the lipid-water interface. The amphipathic segment consisting of residues 689–703 demonstrated distinct parallel positional preference to lipid-water interface. Dominant individual residue contribution was not observed in the segment rearrangement and reorganization, overall residue preference in this rearrangement and reorganization is highlighted.
