# Supplementary material for: Stability, orientation and position preference of the stem region (residues 689-703) in Hepatitis C Virus (HCV) envelope glycoprotein E2: a molecular dynamics study
Source: F1000Res. 2013 Jul 2;2:64. Originally published 2013 Feb 28. [Version 2] doi: 10.12688/f1000research.2-64.v2 (PMC3886794; doi:10.12688/f1000research.2-64.v2)
Supplement: Peer review history

# Peer review: Referee response for version 2

**Published:** 2013-09-03

**Authors:** Gloria Fuentes

**Affiliations:** 1 Bioinformatics Institute, A*STAR, Matrix, Singapore

I appreciate the effort by the authors to improve their draft following the reviewers suggestions. Although they did not address all the points mentioned, I think the current version is more consistent, and few vague comments have been modified. The additional sections provide useful information on the system that improve the understanding of the preference in position and orientation of the amphipathic segment.

Regarding the extension of the simulations, although I would keep insisting in the importance of it, I can understand that the required computer power might not be accessible to everyone. They have included a sentence pointing at this factor, and as far as I am concerned, it should be enough for the acceptance of their work. I am looking forward to the extension in this topic from the authors.

I have read this submission. I believe that I have an appropriate level of expertise to confirm that it is of an acceptable scientific standard.

# Peer review: Referee response for version 2

**Published:** 2013-08-12

**Authors:** Martin Zacharias

**Affiliations:** 1 Physics Department, Technical University Munich, Garching bei München, Germany

I believe the authors made additions and corrections to the original article following the suggestions of the first review report. The article is now acceptable including a discussion on the limitations of MD simulations length and force field approximations.

I have read this submission. I believe that I have an appropriate level of expertise to confirm that it is of an acceptable scientific standard.

# Peer review: Referee response for version 1

**Published:** 2013-04-18

**Authors:** Victor Munoz, Ravishankar Ramanathan

**Affiliations:** 1 Department of Chemistry and Biochemistry, University of Maryland, College Park, College Park, MD, USA; 2 CNB-CSIC, Madrid, Spain

**General Comments and Recommendation: **The title is appropriate, the design of the simulations, and the methods and analysis employed are state of the art. The data provided is sufficient. However, the conclusions are overstretched and much longer simulations are needed to substantiate the claims. 

**More detailed review:**The article describes simulation studies of a 64-residue transmembrane fragment of the envelope glycoprotein E2 domain consisting of two segments: TM (residues 715-746) and ST (residues 683-714). The objective of the study is mainly to understand the role and structural dynamics of the ST segment. MD simulations have been performed on three different starting points: 2 models and one experimentally derived structure. The experimental structure 2KZQ is only 36 residues long itself, lacking the TM segment, and has large dynamics within the NMR structural ensemble (36 models with mean RMSD of 11.32). It is the homolog that produced the best model in I-Tasser, the authors have used in the study. The authors do not mention which model from the NMR ensemble they used in their MD simulations.  Though the authors refer to previous experimental studies that show that the ST segment is helical upon membrane binding, a simple sequence-based helical propensity prediction of the HCV E2 genotype 1a (H77 strain) sequence (Uniprot ID P27958) could be included (to further substantiate their 'ideal helix' model used for the MD simulations).  One of the main results of the article is that the ST segment has a stable helical conformation, stabilized more by the lipid-peptide interactions than the TM-ST peptide-peptide interactions. The similarity between the three different simulations, even with one of them lacking the TM segment, forms the basis of their conclusion. The authors observe uncharacteristic spikes towards the end in the simulations of NMR-structure and the I-Tasser model MD simulations (Figures 2, 3a, 3b). Since the NMR-structure simulation is very short (just 20ns) compared to the I-Tasser model simulation, the authors could perform a longer, equivalent 100ns simulation to compare them and ascertain further the importance of lipid-peptide interactions in the ST helical segment stability. Also, given that in the I-Tasser simulation, there is a lot of dynamics observed after the 20ns timepoint until the end of the simulation, a longer simulation of the NMR-structure is recommended.   The other key result which we found interesting is the orientational and positional preference of the amphipathic ST segment, which seems to be parallel to the lipid leaflets. Reorganization and movement of the ST segment towards the hydrophobic core of the lipids, as shown in Fig S1, is observed clearly in the I-Tasser model simulation, whereas the initial horizontal orientation is maintained in the other simulations. The authors could identify the key residues involved in this re-organization and highlight them

We have read this submission. We believe that we have an appropriate level of expertise to confirm that it is of an acceptable scientific standard, however we have significant reservations, as outlined above.

# Peer review: Referee response for version 1

**Published:** 2013-04-18

**Authors:** Gloria Fuentes

**Affiliations:** 1 Bioinformatics Institute, A*STAR, Matrix, Singapore

The authors have carried out MD simulations in lipid bilayer for three different models for the C-terminal region of the envelope glycoprotein E2. The use of different structures provides *a priori* a good benchmarking for the observations claimed in the article. However, the trajectories presented in this work seem not to have converged completely. The plots will show the trend more clearly if running average is used rather than the raw data. With the current advances in hardware and software, it seems to me that the authors have explored a limited conformational space of the system, which might be masking some other biological conformations for the region in the study.

I am missing a more elaborated discussion in context with the available experimental data that they refer to in the conclusion. So far the correlation done in this study is too vague. The paper with experimental data the authors mentioned also suggests that this amphipathic helix could fold upon lipid binding. A MD simulation in water could help to elucidate this experimental observation. It would be convenient if the authors could specify the exact definition of membrane interface; for those people not working in the field, it could be a bit confusing. This concept could suggest the interface of the phospholipid bilayer (membrane core) or the lipid-water interface.

The legend for the Supplementary Figure is missing the colour code used.

I have read this submission. I believe that I have an appropriate level of expertise to confirm that it is of an acceptable scientific standard, however I have significant reservations, as outlined above.

# Peer review: Referee response for version 1

**Published:** 2013-03-26

**Authors:** Martin Zacharias

**Affiliations:** 1 Physics Department, Technical University Munich, Garching bei München, Germany

The paper describes comparative MD simulations on the E2 TM region embedded in a lipid membrane. Different start conformations have been considered based on different molecular modelling methods + a structure from the pdb. I believe the simulations have been performed properly. Some interesting results on orientational preference of the helices have been reported. However, it should be emphasized that the simulations are too short to achieve convergence of the simulation results. This should be discussed in the Results & Discussion section or in the Conclusion section. Also, comparison to other simulation studies on peptide helices in membranes are missing (e.g. of the Thielemann group). At the end of the conclusion section the authors state “The results also revealed the orientation and positional preferences of the amphiphilic segment in relation to the water-bilayer interface that further clarify speculations from experimental studies.” 

The authors mention “.. speculations from experimental studies.”, however, no reference to such speculations have been given and the details of the speculations are not discussed. The authors should give proper reference and should in detail explain what is meant by their statement.

I have read this submission. I believe that I have an appropriate level of expertise to confirm that it is of an acceptable scientific standard, however I have significant reservations, as outlined above.
